# Vitamin A Levels Among Pre-School Children of Central and Western China

**DOI:** 10.3389/fpubh.2021.694106

**Published:** 2021-09-06

**Authors:** Qian Chen, Yongfang Liu, Li Chen, Jie Chen, Ting Yang, Qian Cheng, Tingyu Li

**Affiliations:** ^1^Department of Child Health Care, Children's Hospital of Chongqing Medical University, National Clinical Research Center for Child Health and Disorders, Ministry of Education Key Laboratory of Child Development and Disorders, Chongqing Key Laboratory of Child Health and Nutrition, Chongqing, China; ^2^Department of Nutrition, Children's Hospital of Chongqing Medical University, Chongqing, China

**Keywords:** vitamin A, pre-school child, Z-score, nutritional status, China

## Abstract

**Objective:** To investigate vitamin A deficiency of pre-school children in central and western China for developing strategies to prevent and control vitamin A deficiency (VAD) among children.

**Design:** From November 2018 to September 2019, a total of 2,194 healthy children aged 2–6 years were enrolled. Serum retinol levels in the children were detected by liquid-phase tandem mass spectrometry. In addition, social demographic and dietary questionnaires were collected through interviews with children's caregivers.

**Setting:** The participants were enrolled in 12 cities or their subordinate jurisdictions in the central and western regions of China.

**Participants:** Two thousand one hundred and ninety four healthy children aged 2–6 years old.

**Results:** Overall, 35.51% (779/2,194) of the children were found to be vitamin A insufficient (VAI, serum retinol < 1.05 μmol/L). Elder children had a higher risk to suffer from VAI, with proportions of 25.00% (87/348), 28.92% (142/491), 38.38% (256/667), and 42.73% (294/688) among children aged 2, 3, 4, and 5 years, respectively. Vitamin A levels were also positively correlated with per capita income (AOR = 1.18) and regional economic level (0.71), and the frequency of milk intake (0.91).

**Conclusions:** The incidence of VAI was higher among children aged 2–6 years, and the incidence of VAI increases with age. VA levels were positively correlated with levels of economic development in the family and region. So prevention strategies for VAD need to focus on pre-school children, especially dairy intake and developing regions.

## Introduction

An essential micronutrient, vitamin A, essentially functions as a vision regulator, anti-infective, maintaining the integrity of epithelial cells, tissue growth and metabolism, and reproductive function (1). Iron and iodine deficiency, in addition to vitamin A deficiency (VAD), account for three major micronutrient deficiency diseases that occur worldwide and influence children's health more significantly (2). About 250 million pre-school children and 19.1 million pregnant women suffered from VAD worldwide as of 2009 (1), which accounts for a significant public health problem in developing countries, where epidemiological studies on the vitamin A nutritional status of children have been conducted (3–6).

VAD causes dryness of the eyes, night blindness, impaired immune system, anemia, and increases mortality in children suffering from measles or diarrhea (7–10). In addition, marginal VAD (MVAD) is far more ignored, although its prevalence is far greater as compared with VAD. MVAD leads to an inadequate vitamin A level, which likely causes anemia, respiratory, and digestive tract infections, besides affecting children's growth and development. The lack of timely intervention results in an easy progression of MVAD to VAD. Thus, early diagnosis of MVAD and interventions to improve its vitamin A level are critical for children's health management. China exhibits a high prevalence of VAD among children and adolescents, caused by factors such as poor economic status and dietary habits, especially in rural areas. The Chinese Nutrition Society, in 2002, conducted a national prevalence survey and reported that 9.3% of children aged 3–12 years were affected by VAD and 45.1% by MVAD, demonstrating a low prevalence of VAD throughout the country (11). However, in remote poor rural areas, including Guizhou, Guangxi, Gansu, Sichuan, Yunnan, etc., the prevalence of VAD was 20% (12). The nutritional status of children and adolescents has improved, as economic conditions have improved in recent years. The prevalence of VAD and MVAD among Chinese children aged 12 years and under was 5.16 and 24.29%, respectively, during 2015 (13, 14). Thereafter, for about 10 years, no large scale epidemiological investigations on vitamin A levels have been conducted in China, owing to which national representative data on vitamin A for children aged 1–6 years are lacking (14).

China exhibits an imbalanced economic status across its vast territory, thus recording variable prevalence of VAD in areas with various economic levels. Regions with a higher prevalence of VAD and whether children of various ages are at the relative risk of developing VAD have not yet been established. Therefore, the vitamin A levels among children aged 2–6 years living in cities of different economic levels and national level impoverished counties of China were studied. Factors influencing vitamin A levels were analyzed to generate a database to formulate strategies to improve vitamin A levels among pre-school children.

## Materials and Methods

### Participants and Sampling Strategy

This study was a multicenter cross-sectional survey. The Ethics Committee of the Children's Hospital of Chongqing Medical University reviewed and approved this study.

The sample size of the study was calculated based on relevant research reports on VAD rates using the following formula: $N=\frac{u_{m}^{2}\;p\;\left(1-p\right)}{\delta^{2}}$. The prevalence of VAD was calculated as 15% (15). Therefore, the probability of class I error was <0.05, and the allowable error between sample prevalence and population prevalence was <5%. In addition, 137 subjects were required for the preliminary study. Considering the 20% non-response rates (missed screening rate), the total sample size for each region should be 172 cases, so we selected about 200 children from each region.

Based on the local economic statistics, cities were segregated into five levels (16): first-level cities comprised municipalities, special administrative regions, and cities with GDP > 160 billion and urban population higher than two million; second-level cities comprised other provincial capitals, sub-provincial cities, special economic zone cities, Suzhou and Wuxi; third-level cities comprised 14 economic-open coastal cities, economically developed, and high-income cities; fourth-level cities comprised other cities with a population of more than one million and key economic cities; and fifth-level cities consisted of other famous economic cities, important transportation hubs, and a key tourist city with a population of more than 500,000.

**Figure 1:**
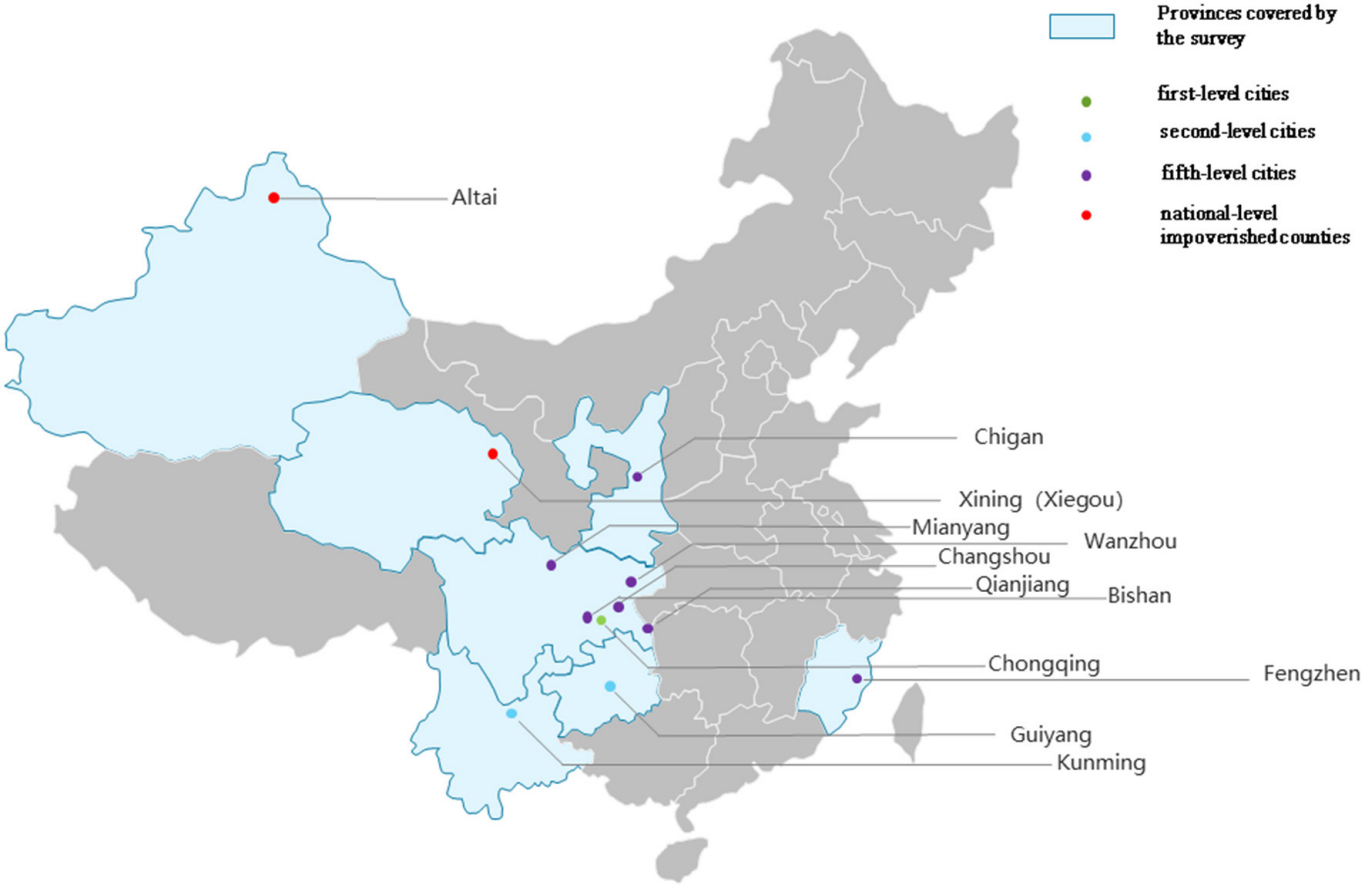
Regions covered by this study.

The central and western regions of China were stratified according to different administrative levels and sorted alphabetically. Finally, the cities were selected by the computer random number extraction method. Twelve cities and counties were selected from among the cities located in the central and western regions of China, comprising a city of the first level, Chongqing; two cities of the second level, Guiyang and Kunming; seven cities of five levels, Mianyang, Bishan, Wanzhou, Changshou, Yongtai, Liquan, and Qianjiang; and two impoverished counties of national level, Datong County of Qinghai and Jimunai County of Xinjiang (Figure 1). Between 172 and 200 samples were expected to be selected from each region.

A cross-sectional study was conducted to enroll healthy children aged 2–6 years who reside in the zones mentioned above. Random selection of children of appropriate ages was carried out from randomly selected kindergartens was performed; hospitals in Wanzhou, Mianyang, Qianjiang and Kunming also enrolled eligible participants from their respective societies.

The inclusion criterion was as follows: (a), 2–6 years old healthy; (b) their parents signed written informed consent. Exclusion criteria were the following: (a) blood levels of C-reactive protein are elevated; (b) chronic unexplained diarrhea, active bleeding from chronic enteritis, severe infection, and gastrointestinal neoplasms; (c) complicated with serious cardio-cerebrovascular, liver, kidney, nerve, and mental diseases; (d) patients who had participated in clinical trials or had been diagnosed with iron deficiency anemia and received iron supplements within the previous 3 months; (e) patients who had received blood transfusions within the previous 4 weeks; (f) children of parents suffering from severe chyloemia or hemolysis sampling were not included in the study. Severely hemolyzed or contaminated and inadequate blood samples were also excluded from the analysis.

### Indicators and Measurements

Trained nurses used structured questionnaires to collect demographic data for children, including ethnicity, occupation of the parents, family income, disease status, diet, nutritional supplementation, and others.

In the diet survey, information on the frequency of the diet was collected through interviews with their parents or caregivers. The food categories include dairy or dairy products; Cereals, tubers, and miscellaneous beans; Vegetables; Fruit class; Eggs; Fish, shrimp, and shellfish; Livestock and poultry meat; Soybeans and soy products; Fat (cooking oil). The survey included the average number of times a week and the average daily amount of these foods the children ate over a period of 3 months. In addition, the nutrient supplementation was investigated by the questions “Did your child take vitamin A or preparations containing vitamin A in the last 3 months? What is the dose?”

Professionals from the pediatric care department measured these children's body weight and height in the morning with a weighing scale and height rod. The children were asked to remove their coats while being measured. The weight readings were accurate to 0.05 kg and those of height to 0.1 cm. Z-scores of weight-for-age (WAZ), height-for-age (HAZ), and weight-for-height (WHZ) were calculated utilizing anthro 2005, based on the World Health Organization (WHO) growth standards for children <5 years of age (17). The children's abnormal nutritional status included malnourished and overnourished. Malnourished was defined as “stunting,” “underweight,” and “wasting,” where HAZ, WAZ, or WHZ is more than two times the standard deviation (SD) less than the population reference, respectively. And children were defined as overnourished if their WHZ was more than two times the SD of the reference, including “overweight” (reference + 2SD < WHZ reference + 3 SD); and “obese” (WHZ > reference + 3SD).

Four milliliter of postprandial venous blood was collected from the children in the morning, serum was segregated by centrifugation, and a third party company quantified vitamin A levels with the use of liquid-phase tandem mass spectrometry (AB SCIEX Framingham, MA, USA). A triple quad mass spectrometer equipped with an Agilent Zorbax Eclipse XDB C8 guard column was used in the testing.

The nutritional status of vitamin A of children was defined per WHO recommended standards (18) as follows: (1) VAD: serum retinol concentration < 0.70 μmol/L; (2) MVAD: Serum retinol concentration is in the range 0.70–1.05 μmol/L; (3) normal vitamin A (VAN): Serum retinol concentration ≥ 1.05 μmol/L; and (4) Vitamin A insufficiency (VAI): Serum retinol < 1.05 μmol/L.

### Data Processing and Statistical Methods

A delineated testing center in each area carried out all the tests, and the data were double entered into Excel 2007 software (Microsoft, Redmond, USA). Continuous data were expressed as mean ± SD, and Student's *t*-test was used for independent samples to compare differences between groups, while comparisons between multiple groups were performed using one-way ANOVA. A Chi-square-test was used to test the proportions between groups. The correlations between variables were analyzed by linear regression. All variables were introduced into the multivariate linear regression model for evaluation after adjustment for confounders such as height, weight, and gender. A stepwise selection technique was applied to include variables with SLE > 0.10 and exclude those with SLS < 0.05. All statistical analyzes were performed in SAS9.4 software (SAS Institute, Cary, NC, USA). *P*-values < 0.05 were considered statistically significant.

## Results

### Basic Descriptions of Participants

In total, 2,194 children aged 2–6 years were enrolled in this study from November 2018 to September 2019 in China. Among them, 1,166 (53.1%) were boys, and 1,028 (46.9%) were girls, and their average age was 3.83 ± 1.14 years. Most enrolled children lived in economically underdeveloped regions, including 1,290 (58.80%) resided in five-level cities and 351 (16.00%) in national level impoverished counties. The age distribution and economic status of the families or areas did not present a statistical difference between men and women, as shown in Table 1.

**Table 1:** Basic demographic and physical characteristics of participants.

	**Total**	**Male**	**Female**	***Z*** **or χ^2^ statistics**	***p*** **-value**
Age (years)	3.83 ± 1.14	3.81 ± 1.14	3.84 ± 1.15		
Age group, *n* (%)	*n* = 2,194	*n* = 1,166	*n* = 1,028	0.813	0.846
2 years old	348	186 (15.95)	162 (15.76)		
3 years old	491	265 (22.73)	226 (21.98)		
4 years old	667	359 (30.79)	308 (29.96)		
5–6 years old	688	356 (30.53)	332 (28.47)		
Locations, *n* (%)	*n* = 2,194	*n* = 1,166	*n* = 1,028		
First-level cities	152 (6.93)	80 (3.65)	72 (3.28)		
Second-level cities	401 (18.28)	208 (9.48)	193 (8.80)	0.756	0.860
Fifth-level cities	1290 (58.80)	685 (31.22)	605 (27.58)		
Poverty counties	351 (16.00)	193 (8.8)	158 (7.20)		
Per capita household income^*^, *n* (%)	*n* = 1,560	*n* = 837	*n* = 2,194		
<500 CNY	176 (11.28)	104 (6.67)	72 (4.62)		
500–999 CNY	223 (14.29)	112 (7.18)	111 (7.12)	0.460	3.622
1,000–2,999 CNY	477 (30.58)	250 (16.03)	227 (14.55)		
3,000–4,999 CNY	363 (23.27)	195 (12.50)	168 (10.77)		
≥5,000 CNY	321 (20.58)	176 (11.28)	145 (9.29)		
**Physical measures^a^**					
HAZ	−0.10 ± 1.58	−0.12 ± 1.57	−0.06 ± 1.61	−0.62	0.537
WAZ	0.07 ± 1.18	0.11 ± 1.21	0.04 ± 1.15	1.27	0.206
WHZ	0.19 ± 1.10	0.27 ± 1.18	0.11 ± 1.00	2.75	0.006
Stunting	93 (6.47)	52 (6.70)	41 (6.20)	0.146	0.702
Underweight	44 (3.06)	27 (3.48)	17 (2.57)	0.990	0.320
Wasting	32 (2.23)	19 (2.45)	13 (1.97)		
Overweight	51 (2.58)	22 (2.84)	15 (2.27)	1.506	0.681
Obese	14 (0.97)	9 (1.16)	5 (0.76)		

* *Data of family income were missing for 634 participants*.

a *437 children had data of physical measures*.

*HAZ, height for age Z-score; WAZ, weight-for-age Z-score; WHZ, weight-for-height Z-score*.

Among the 1,437 (65.50%) children who were measured for height and weight, the height-for-age (HAZ) and weight-for-age (WAZ) Z scores were similar between boys and girls, while the weight-for-height (WHZ) Z scores were higher among boys (0.27 ± 1.18) than among girls (0.11 ± 1.00) (*p* = 0.006). The proportions of malnourished and overnourished children were similar between boys and girls, with 128 (8.91%) malnourished children including 93 (6.47%) stunting, 44 (3.06%) underweighted, and 32 (2.23%) wasting children, and 51 (3.55%) overnourished children including 37 (2.58%) overweight and 14 (0.97%) obesity (Table 1).

### Prevalence of VAD Prevalence Among Children of Various Ages and Economic Status

**Figure 2:**
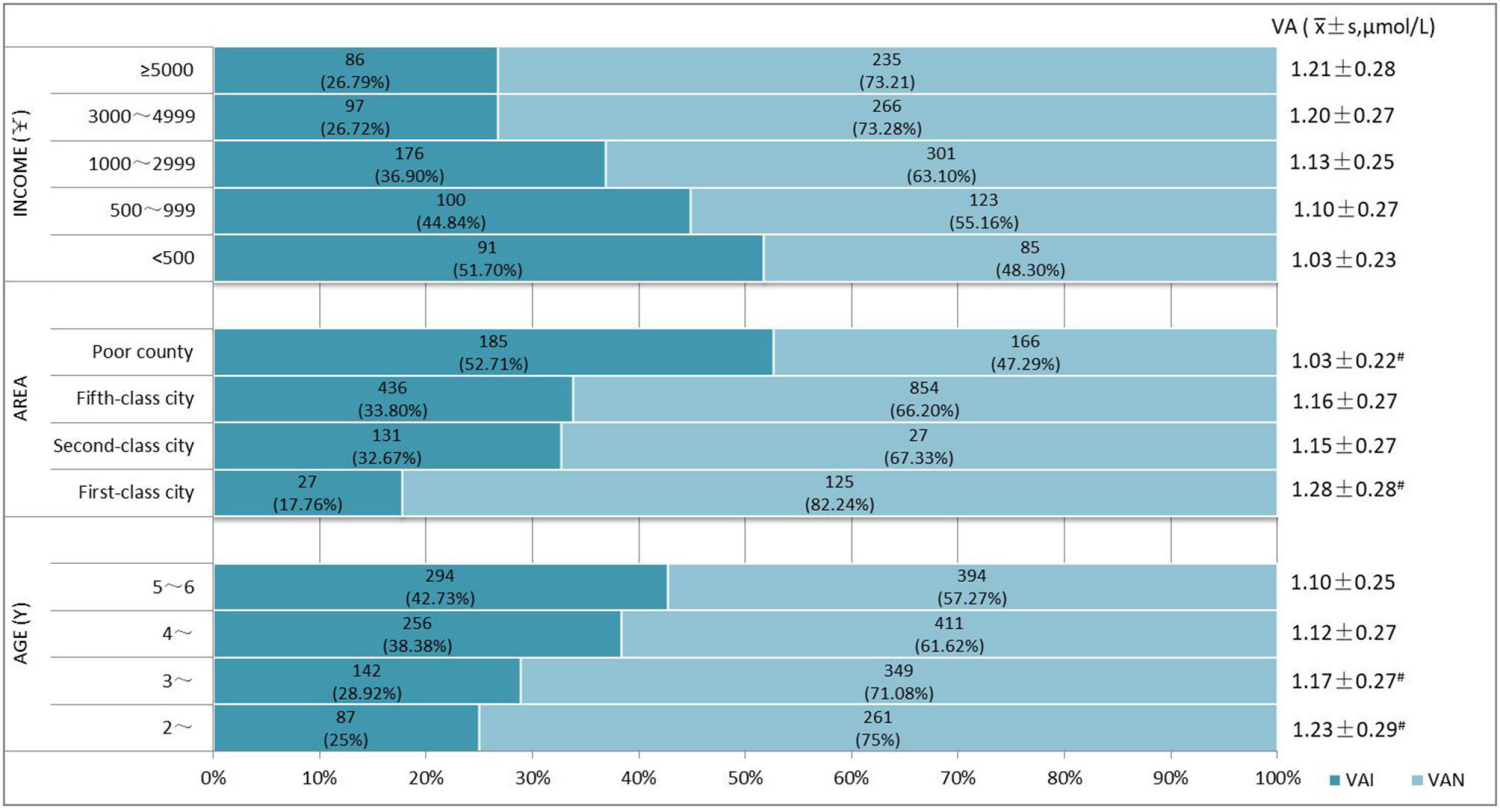
Vitamin A levels and prevalence of vitamin A insufficiency (VAI) in different groups. VAD, vitamin A deficiency, serum retinol < 0.70 μmol /L; MVAD, marginal vitamin A deficiency, 0.70 μmol /L ≤ serum retinol < 1.05 μmol /L; VAN, normal vitamin A, serum retinol ≥ 1.05 μmol/L; #Significant difference in vitamin A levels and prevalence of VAD and MVAD between groups (*p* < 0.05).

Overall, blood samples from 2,194 children have been certified for vitamin A quantification. Crudely, serum vitamin A of Chinese pre-school children was averagely 1.15 ± 0.27 μmol/L, of whom 35.51% (779/2,194) were determined as VAI, including 2.64% (58/2,194) as VAD and 32.86% (721/2,194) as MVAD. Serum vitamin A levels among children were found to increase with increasing age, and older children were found to have a higher risk of having VAI (Figure 2). Children aged 5–6 years had the highest proportions (42.73%, 294/688) of VAI, followed by 38.38% (256/667) among children of 4-year-olds, 28.92% (142/491) of 3-year-olds and 25.00% (87/348) of 2-year-olds (Figure 2).

**Figure 3:**
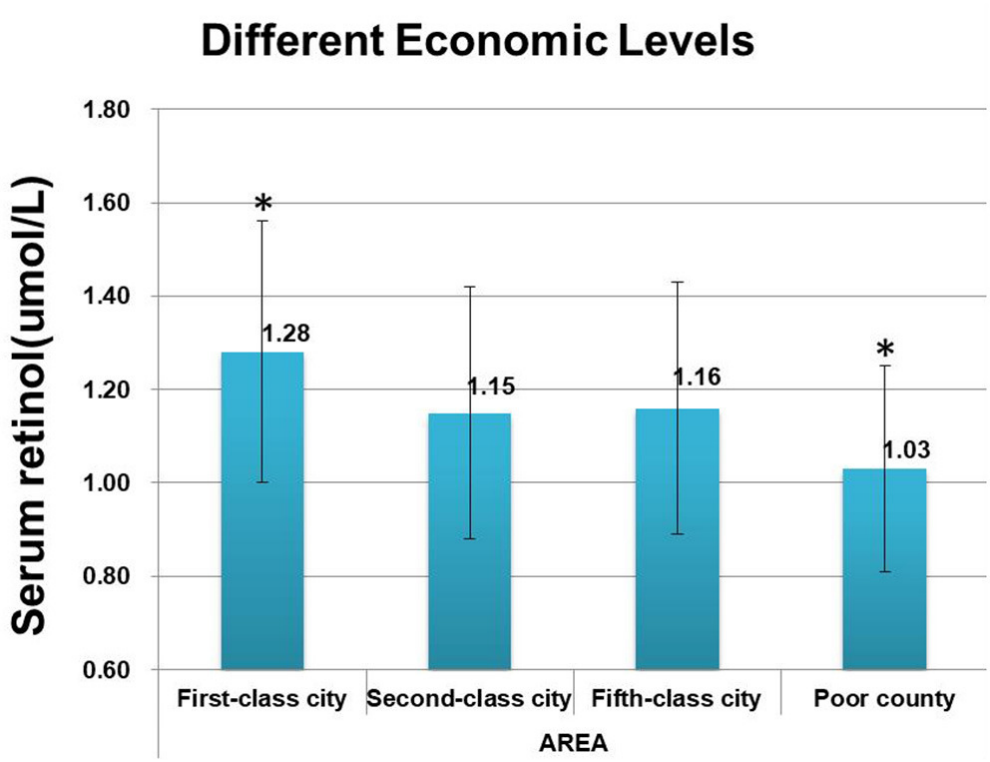
Comparison of vitamin A levels in cities of different economic levels. *Significant difference from other groups (*p* < 0.05).

**Figure 4:**
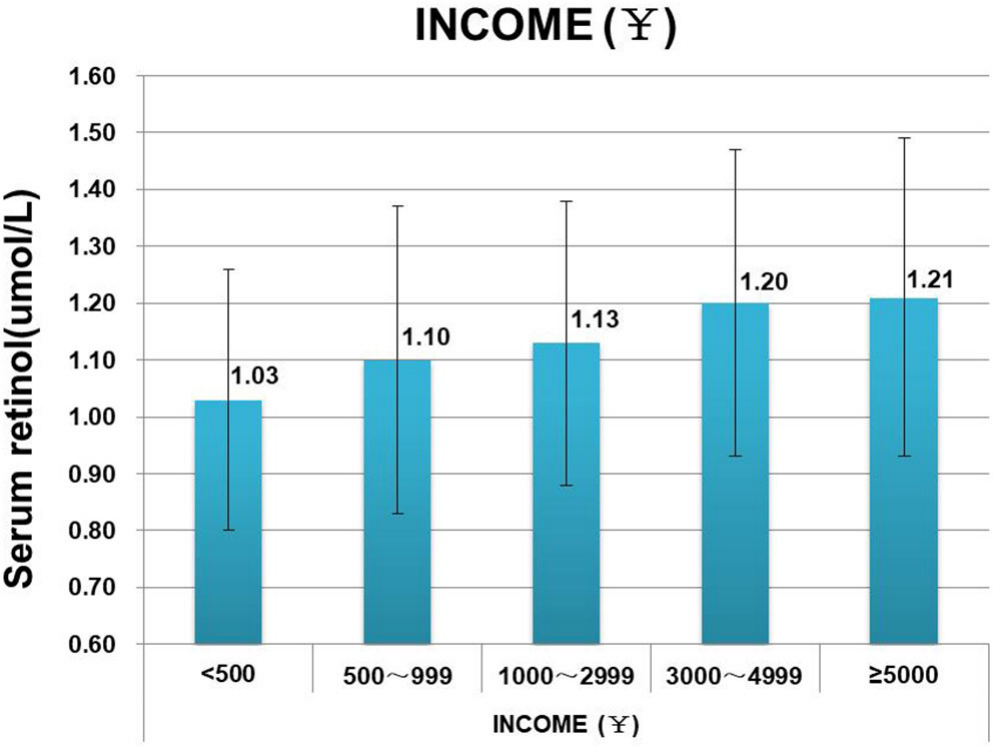
Comparison of vitamin A levels of children from households of different income.

**Figure 5:**
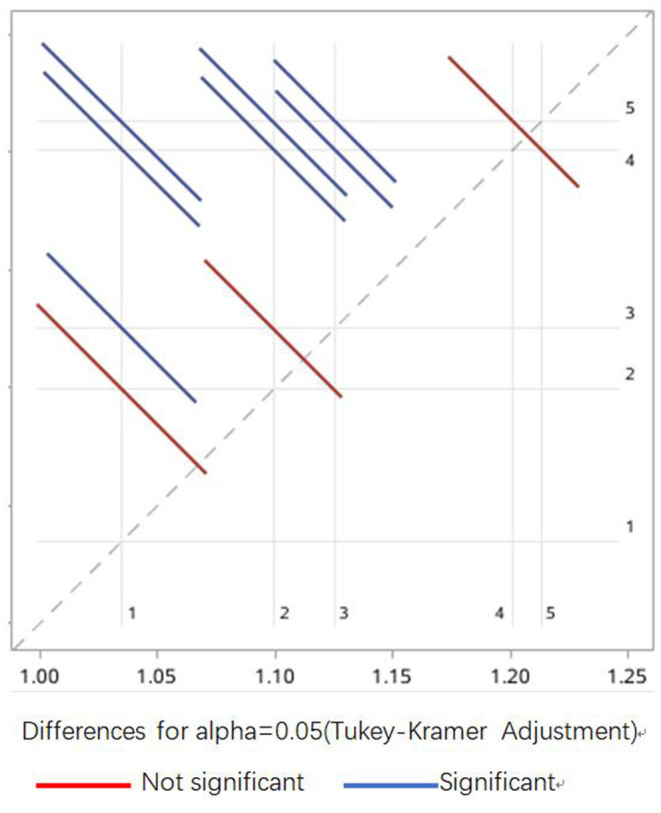
Statistical difference between Vitamin A levels of children from households of different income. 1 ≤ 500 CNY, 2 = 500–999 CNY, 3 = 1,000–2,999 CNY, 4 = 3,000–4,999 CNY, 5 ≥ 5,000 CNY. The blue lines indicate a significant difference between the paired groups, and the red lines indicate no differences between the paired groups.

Meanwhile, VA levels increased with increasing regional economy and household income (Figure 3). The prevalence of VAI among children in impoverished counties at the national level (50.71%, 185/351) was remarkably higher than in other areas (*p* < 0.001). Children in first-level cities exhibited the lowest VAI prevalence of 17.16% (27/152), and the VAI prevalence was comparable between children who lived in cities of the second and fifth level [32.67% (131/401) vs. 33.80% (436/1,290)]. The prevalence of VAI prevalence was found to decrease with increasing household income, and the proportions of children suffering from VAI were estimated to be 51.70% (91/176), 44.84% (100/223), 36.90% (176/477), 26.72% (97/363), 26.79% (86/321) in the household with a per capita income of <500 CNY, 500~999 CNY,1,000~2,999 CNY, 3,000~4,999 CNY, and >5,000 CNY, respectively, and statistical significance by the Chi-square test for trend (*p* < 0.0001). No significant differences were observed in the incidence of VAI among children from families with incomes of 3,000–4,999 CNY and 5,000 CNY. VA levels were observed to increase with increasing family income (Figure 4). And the differences between some groups were statistically significant (*p* < 0.05, Figure 5).

### Supplementation of Vitamin a Among Children of Different Age Groups

To examine the effect of vitamin A supplementation on children's nutritional level, we also asked whether vitamin A supplements were administered to the children. Overall, 5.41% (104/1,921) of children aged 2–6 years were given vitamin A supplements. Higher percentages of children aged 2–3 years (11.29%, 35/310) and aged 3–4 years (6.70%, 30/448) were administered vitamin A supplements, compared with children aged >4 years (3.35%, 39/1,163). Regardless of whether the children took vitamin A supplements, 5.41% (104/1,921) and 94.59% (1,818/1,921) suffered VAI with a significant difference (*p* = 0.009) (Table 2).

**Table 2:** Supplementation of vitamin A in children of different age groups (*n* = 1,291).

**Age group (years)**	**Yes, *n* (%)**	**No, *n* (%)**	***x*** ^**2**^	***p***
2~	35 (11.29)^*^	275 (88.71)		
3~	30 (6.70)^*^	418 (93.30)	32.00	<0.0001
4~	19 (3.28)	560 (96.72)		
5~6	20 (3.42)	564 (96.58)		
Total	104 (5.41)	1817 (94.59)		

* *Differences were statistically significant compared to the other groups (p < 0.05)*.

### Risk Factors for VAD Among Pre-School Children

After adjusting for the variables of gender, height, and weight comprising the per capita household income, potential significant variables were introduced into the stepwise multivariate linear regression analysis, including the quantity of eggs and milk intake, age, economic level of the residing region, and vitamin A supplementation. Table 3 shows the outcome of the analysis. Vitamin A in children aged 2 to 6 years was found to be positively correlated with an increase in milk intake frequency (adjusted OR = 0.91) and among children of the family in (adjusted OR = 1.18), or those who reside in a more economically developed area (adjusted OR = 0.71). All of the above differences were statistically significant (*p* < 0.05).

**Table 3:** Multiple stepwise regression analysis of the VA level (*n* = 1,475).

**Parameter**	***B***	**Standard error**	**Wald Chi-Square**	***P***	**OR**	**95%CI**
Income	0.33	0.04	14.49	<0.0001	1.18	(1.08, 1.28)
Milk intake	0.10	0.04	4.86	0.028	0.91	(0.83, 0.99)
Age	−0.29	0.05	28.33	<0.0001	0.76	(0.69, 0.84)
Location	−0.40	0.09	15.67	<0.0001	0.71	(0.60, 0.84)

## Discussion

This multicenter cross-sectional VAD prevalence survey enrolled 2,194 pre-school children aged 2–6 years in 12 cities or their jurisdictions from eight provinces of underdeveloped central and western regions of China. 2.64 and 32.86% of the investigated children were found to have VAD and marginal VAD (MVAD), respectively, which was much lower than that reported in the last national survey in 2002 (9.3% for VAD and 45.1% for MVAD) (11). These data indicate that VAD remains a lower-level public health concern in China according to WHO classification (1, 19). Although the prevalence of VAD is at a low level, many children remain under marginal vitamin A deficiency (20, 21).

Overall, WAZ, HAZ, and WHZ scores for children aged 2–6 years were recorded to be lower than the national level reported in 2015 (22), which can be attributed to the high proportion of surveyed families who reside in poor counties or fifth-tier cities and lower (proportion of 74.79%). The prevalence of malnutrition among children in these regions is higher than among children in urban areas. Nevertheless, the incidence of abnormal physical growth, including rates of malnutrition, were all estimated to be lower than those at the national level in 2015 (stunting 14.3%; underweight 7.8%; wasting 2.5%), so as the rates of overnutrition (overweight 3.4 and obesity 2.0%) (11, 23). Although children's growth level in the central and western regions is still lower than that at the national level (23–25), the incidence of extreme physical conditions is found to be low, and the prevalence of widespread nutritional deficiency diseases among children is currently lower than before (26).

This study explored many areas and covered multiple cities and counties of various economic levels in central and western China. Therefore, the results represent and describe the current levels of vitamin A among children in underdeveloped rural areas of China. Vitamin A levels among children in first-level cities were remarkably higher than those of children in other areas, and the prevalence of VAD was only 1.32%, suggesting a relatively lower prevalence of VAD in economically developed cities. However, in impoverished national counties, the prevalence of VAD among children was up to 3.99%, and the prevalence of VAI was found to have increased to 52.71%, evidencing the current poor nutritional status of vitamin A among children in impoverished counties of China. We believe that different groups of people should consider giving different vitamin A supplementation strategies (27). Policies, related sources and guidelines are an urgent pre-requisite to ensure the improvement of vitamin A nutrition in impoverished counties.

Through this study, a declining trend in vitamin A levels has been observed among children of increasing age, while some other studies reported that younger children had lower vitamin A levels than elder children (14, 28). Elder children were considered to take an abundant diet, including foods rich in vitamin A, which renders them at a lower risk of suffering from VAI. Nevertheless, in this study, parents were found to pay greater attention to the intake of a balanced diet when children were younger-−70.51% (507/719) of children aged <4 years consumed milk every day, compared with 47.22% (527/1,116) of children aged ≥ 4 years. The amount of milk intake also decreases gradually with age, which is fortified with vitamin A. With increasing age, the frequency and quantity of daily egg intake were not changed significantly. Parents were recommended to provide their children with vitamin A supplements, but several parents found it unnecessary and stopped supplementation as their children grew up. For children aged 2–6 years, vitamin A intake through the daily diet is insufficient, while vitamin A stored in the body is gradually utilized, leading to a lower level of vitamin A among elderly children. The problems of the nutritional status of pre-school children have been discussed here, and the need to take vitamin A supplements in addition to the daily diet is enhanced. Furthermore, in the multiple regression analysis, no statistical differences were observed between egg intake and vitamin A supplementation and VAI. This may be related to the following factors. First, in the food frequency table, the egg intake can only be accurate to “<1/1–2 eggs per day,” which cannot be more accurate, leading to little difference in egg intake among different groups. Therefore, in the multivariate regression analysis, it has a low weight and cannot be shown in the results. Second, the proportion of vitamin A supplementation was very small, only about 100 cases, which was statistically significant between different groups in the separate analysis. However, due to the low weight in the multiple regression analysis, there could be no difference in the final results. Detailed investigations of daily dietary intake can present more evidence of dietary intake on the improvement of children's health.

## Conclusions

To summarize, this cross-sectional survey of vitamin A levels among pre-school children in central and western China found a relatively higher level of VAD in economically underdeveloped areas compared to developed cities. The incidence of VAI was higher among children aged 2–6 years, and the incidence of VAI increases with age. As a consequence, policies to improve vitamin A nutrition must be improved among children residing in impoverished counties, including nutrition education and improved vitamin A and related foods supplements.

## Data Availability Statement

The raw data supporting the conclusions of this article will be made available by the authors, without undue reservation.

## Ethics Statement

The studies involving human participants were reviewed and approved by Ethics committee of the Children's Hospital of Chongqing Medical University. Written informed consent to participate in this study was provided by the participants' legal guardian/next of kin.

## Author Contributions

QChen collected the data, validated the statistical results, and wrote the initial draft of the manuscript. TL and QCheng conceived, designed the study, and confirm the authenticity of all the raw data. QCheng, JC, and TY participated in the logistical planning of the study. YL provided statistical support for the sample size estimates and the design of the statistical analysis. LC modified the chart of the article. All authors read and approved the final manuscript.

## Conflict of Interest

The authors declare that the research was conducted in the absence of any commercial or financial relationships that could be construed as a potential conflict of interest.

## Publisher's Note

All claims expressed in this article are solely those of the authors and do not necessarily represent those of their affiliated organizations, or those of the publisher, the editors and the reviewers. Any product that may be evaluated in this article, or claim that may be made by its manufacturer, is not guaranteed or endorsed by the publisher.
